# Supplementary figures and images for: Within-host rhinovirus evolution in upper and lower respiratory tract highlights capsid variability and mutation-independent compartmentalization
Source: bioRxiv. 2023 May 11:2023.05.11.540440. Preprint. [Version 1] doi: 10.1101/2023.05.11.540440 (PMC10197658; doi:10.1101/2023.05.11.540440)

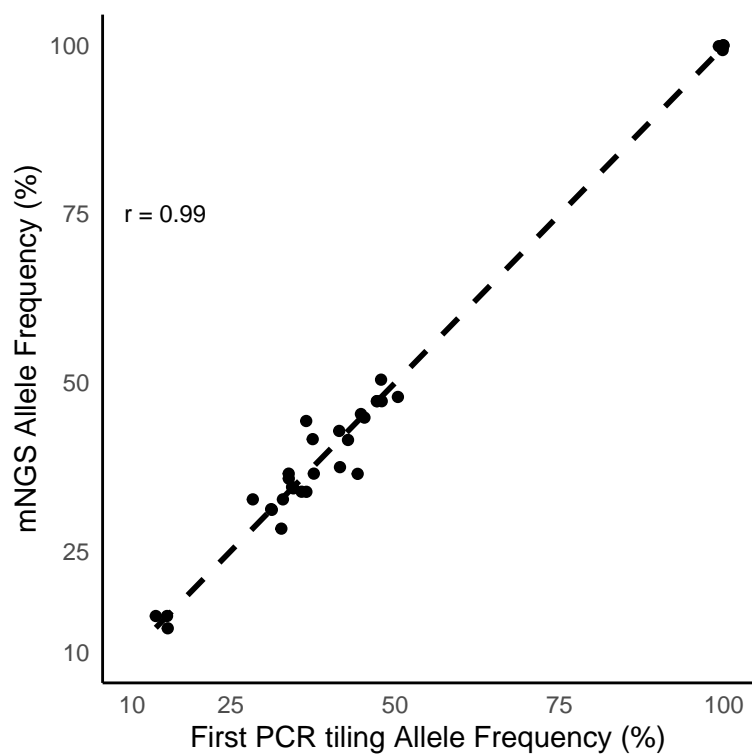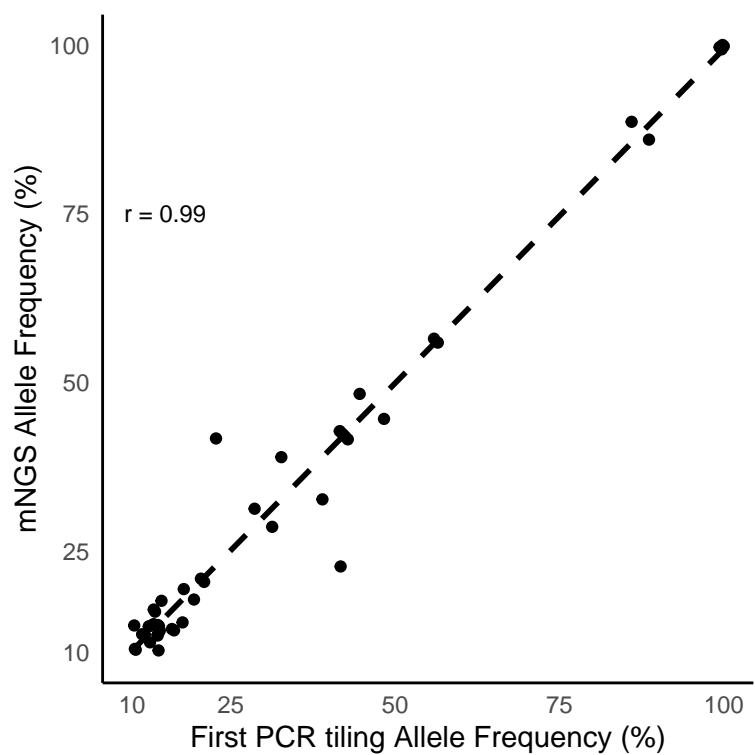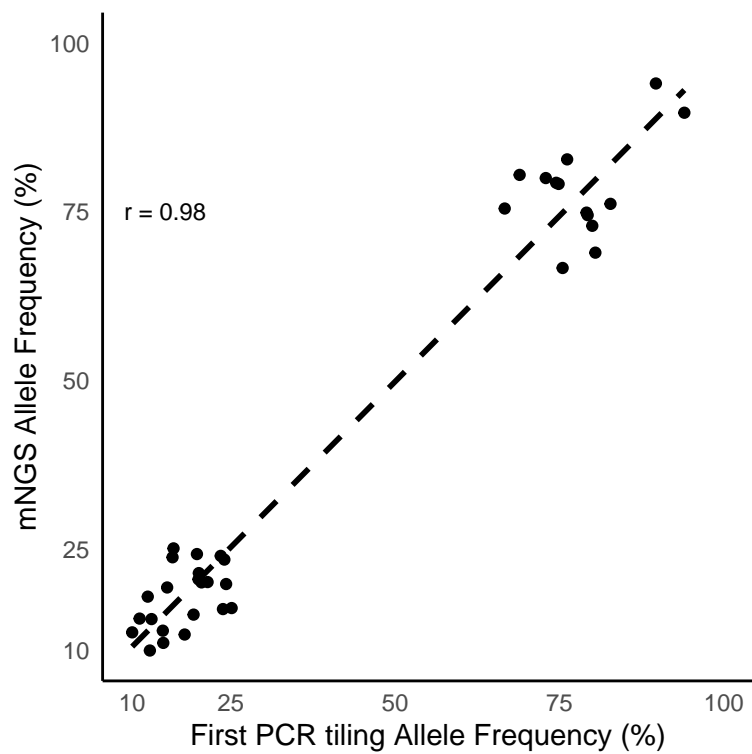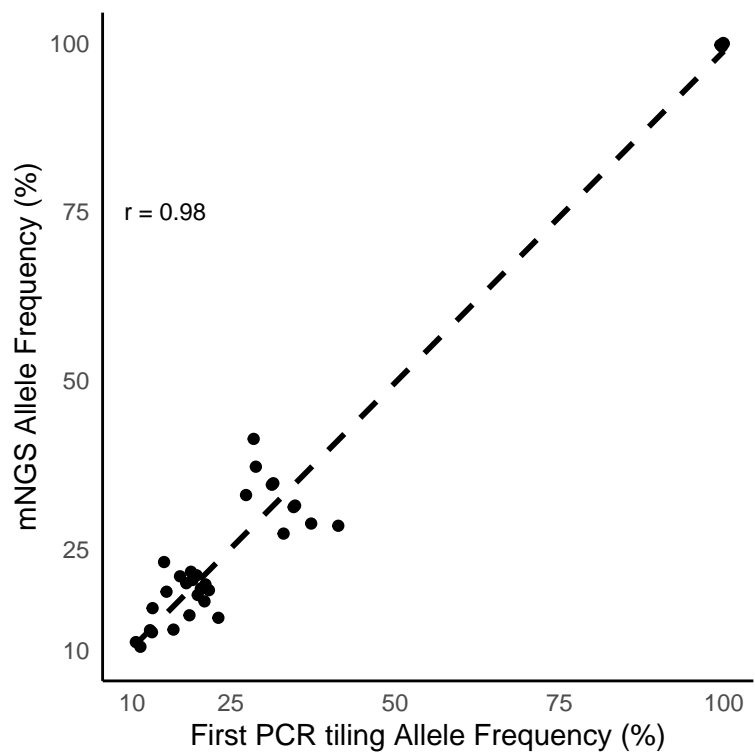

Supplement: Supplement 5 — Supplemental Figure 1. Comparison of iSNV detection by amplicon-based and metagenomic sequencing. Scatter plots show the allele frequency (in percentage) of a given iSNV found in sample BAL11b during different rounds of amplicon-based sequencing (A) and among both amplicon-based and metagenomic sequencing for samples NW11 (B), BAL4 (C) and NW3 (D). [file media-5.pdf]

HRV serotype

- HRV-A
- HRV-B
- HRV-C

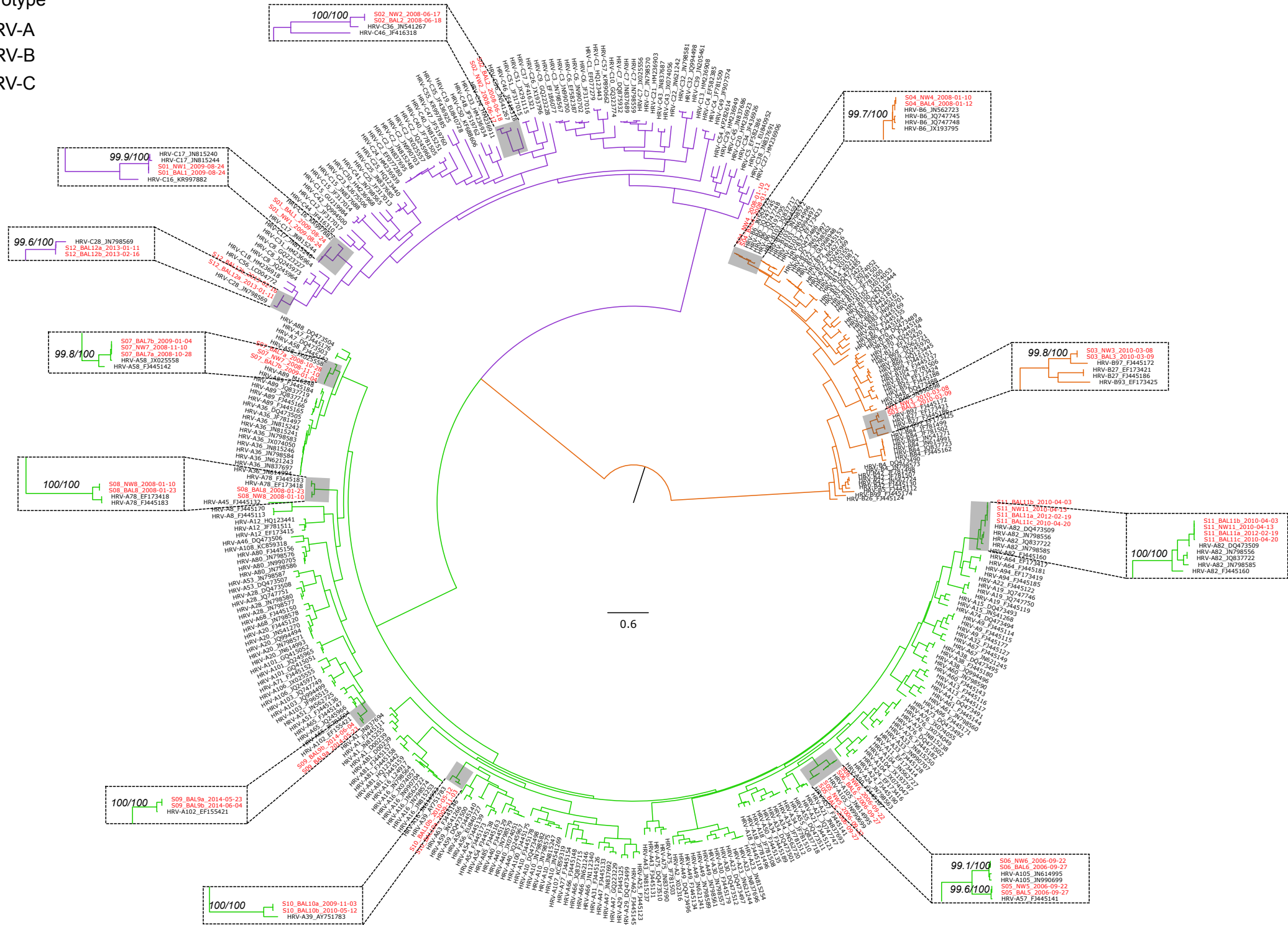

Supplement: Supplement 6 — Supplemental Figure 2. Phylogenetic classification of HRV samples. Maximum likelihood inference of the VP1 nucleotide sequence including reference sequences of all HRV-A, HRV-B and HRV-C serotypes. The name of the consensus sequences for HRV from each sample is highlighted in red. The HRV serotype in each reference is informed in the tip name. Statistical support of relevant phylogenetic clades’ association (SH-alrt / UF-Bootstrap) are indicated. HRV-A, HRV-B and HRV-C clades are denoted with the branch colors. [file media-6.pdf]

**A**

Patient S02, HRV-C39

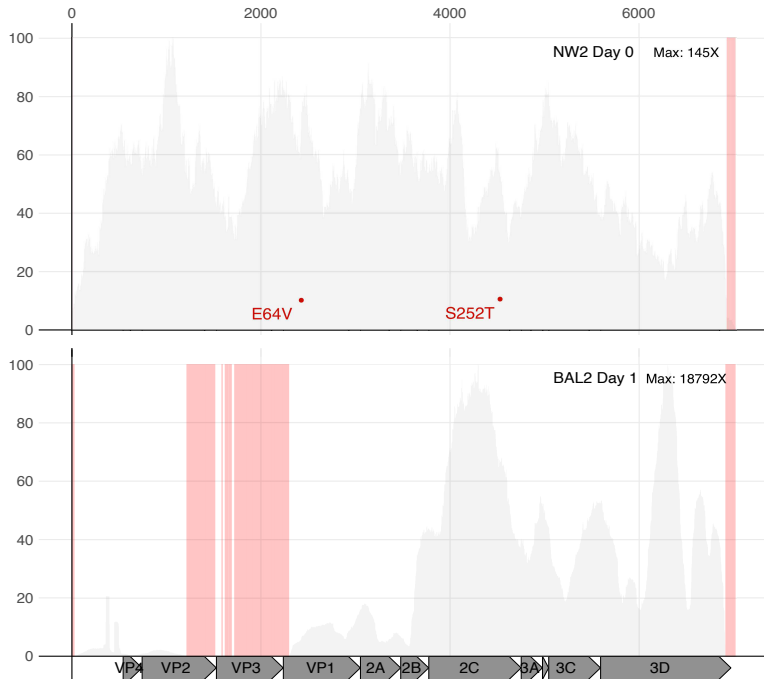**B**

Patient S06, HRV-A105

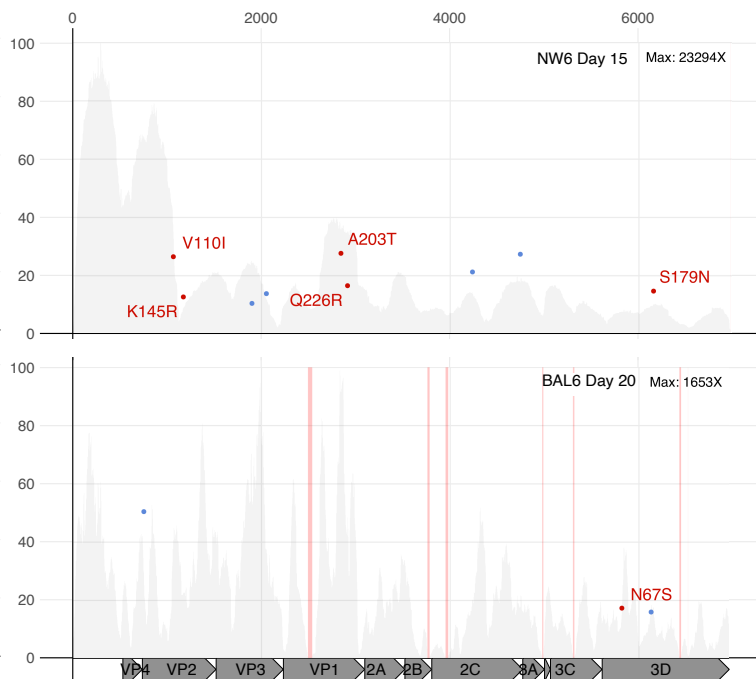

Supplement: Supplement 7 — Supplemental Figure 3. HRV evolution in upper and lower respiratory tract samples comparison at short infection times in immunocompromised individuals. Minor allele frequency (in percentage) of each iSNV across the HRV genome in individuals S02 (A) and S06 (B) is represented by dots. Blue dots indicate iSNV causing synonymous mutation and red dots indicate non-synonymous mutation which also includes the amino acid change description. Upper (NW, nasal wash) and lower (BAL, bronchioalveolar lavage) respiratory tract samples are detailed in the upper right of each plot. In addition, the day when the sample was collected regarding to the first HRV positive and the maximum sequencing depth of coverage for each sample is informed. HRV secondary proteins annotation are indicated at the bottom of the graphics. Grey plot at the background informs the sequencing coverage profile for each sample. Pink-colored regions denotes low sequencing coverage, thereby not analyzed. [file media-7.pdf]

**A**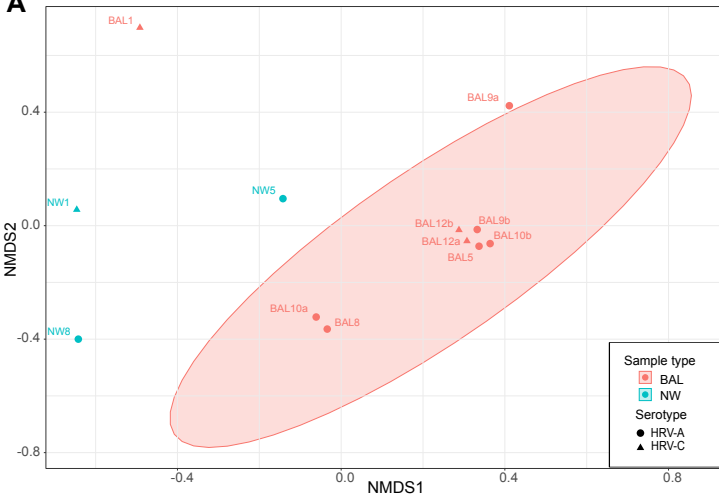**B**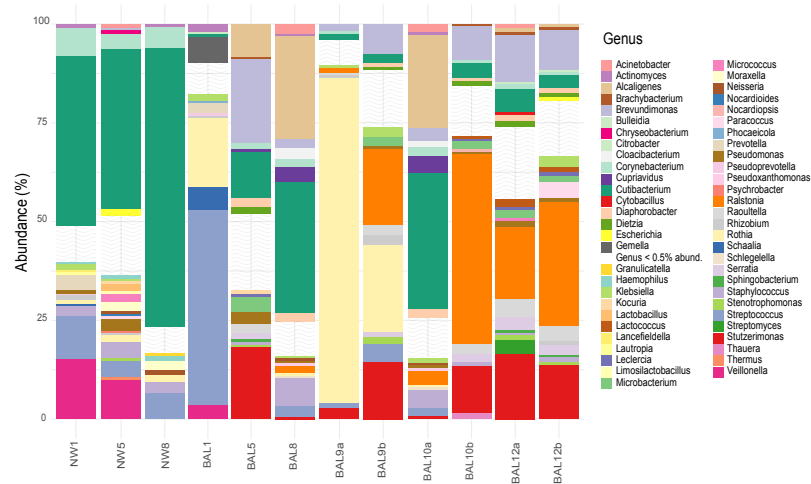

Supplement: Supplement 8 — Supplemental Figure 4. Microbiome characterization in upper and lower respiratory tract samples from immunocompromised individuals during HRV infection. A) NMDS ordination with Bray-Curtis dissimilarity matrix based on the relative abundance of bacteria genus communities. Ellipse of 95% confidence level for the multivariate t-distribution were constructed by using the ‘stat_ellipse’ function of ggplot2 package to illustrate the lower (BAL) respiratory tract samples. No 95% ellipse could be estimated for upper (NW) respiratory tract samples due to the low number of samples. B) Bar-plots show the relative abundance of bacterial genus found in each sample. [file media-8.pdf]

Mutation: • Non Synonymous  
• Synonymous

**A**

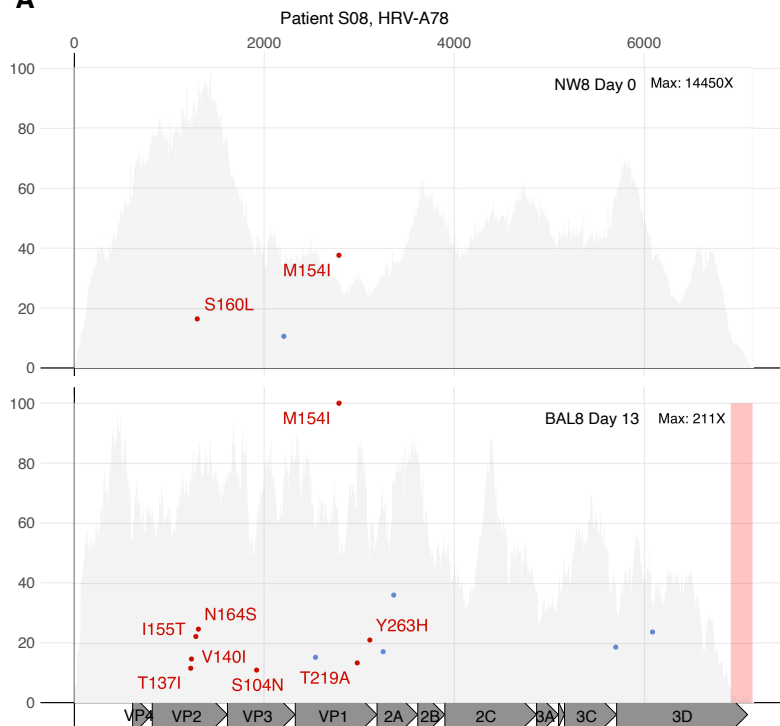

**B**

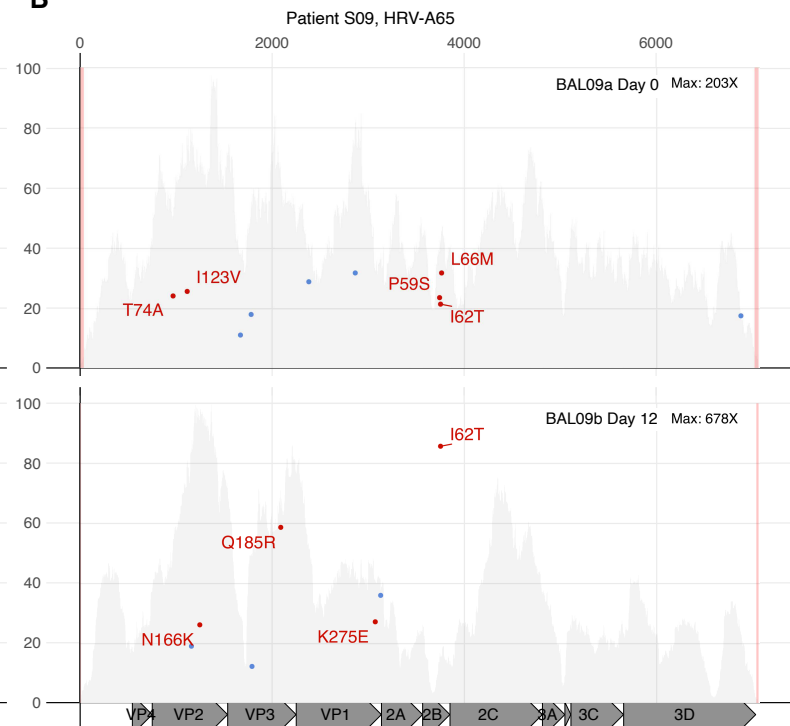

**C**

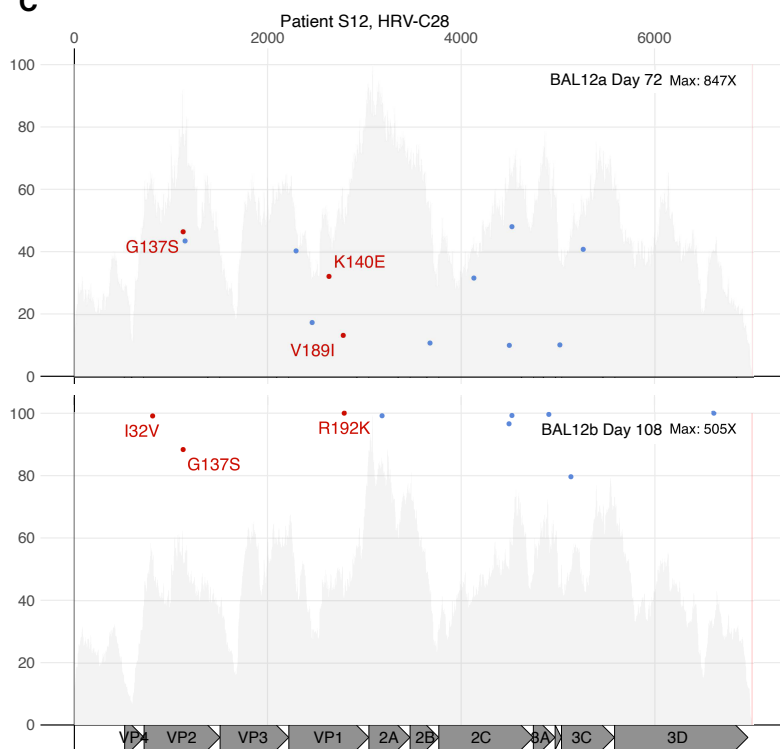

Supplement: Supplement 9 — Supplemental Figure 5. Long-term HRV infection dynamics in immunocompromised individuals. Minor allele frequency (in percentage) of each iSNV across the HRV genome in individuals S08 (A), S09 (B) and S12 (C) is represented by dots. Blue dots indicate iSNV causing synonymous mutation and red dots indicate non-synonymous mutation which also includes the amino acid change description. Upper (NW, nasal wash) and lower (BAL, bronchioalveolar lavage) respiratory tract samples are detailed in the upper right of each plot. In addition, the day when the sample was collected regarding to the first HRV positive and the maximum sequencing depth of coverage for each sample is informed. HRV secondary proteins annotation are indicated at the bottom of the graphics. Grey plot at the background informs the sequencing coverage profile for each sample. Pink-colored regions denotes low sequencing coverage, thereby not analyzed. [file media-9.pdf]

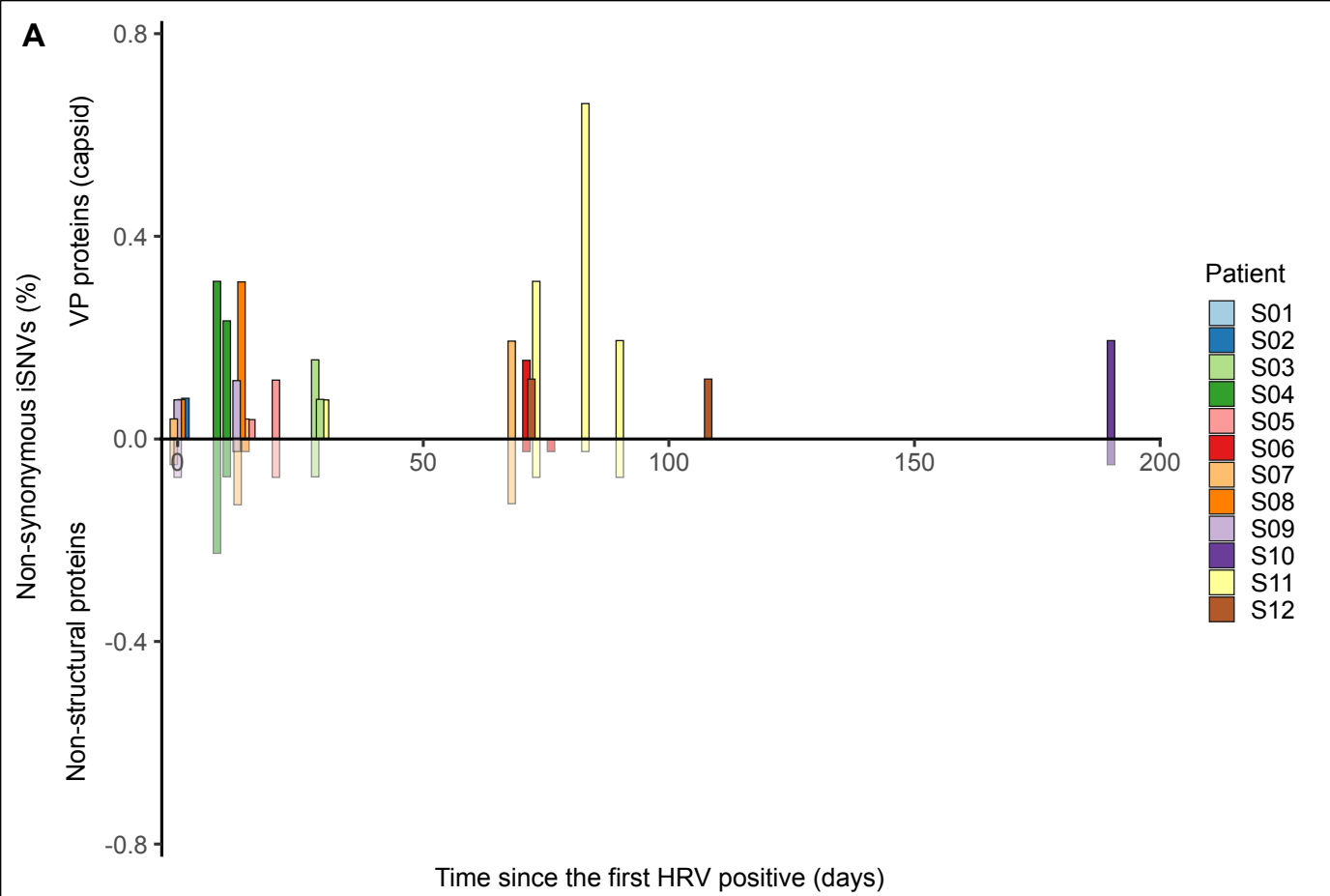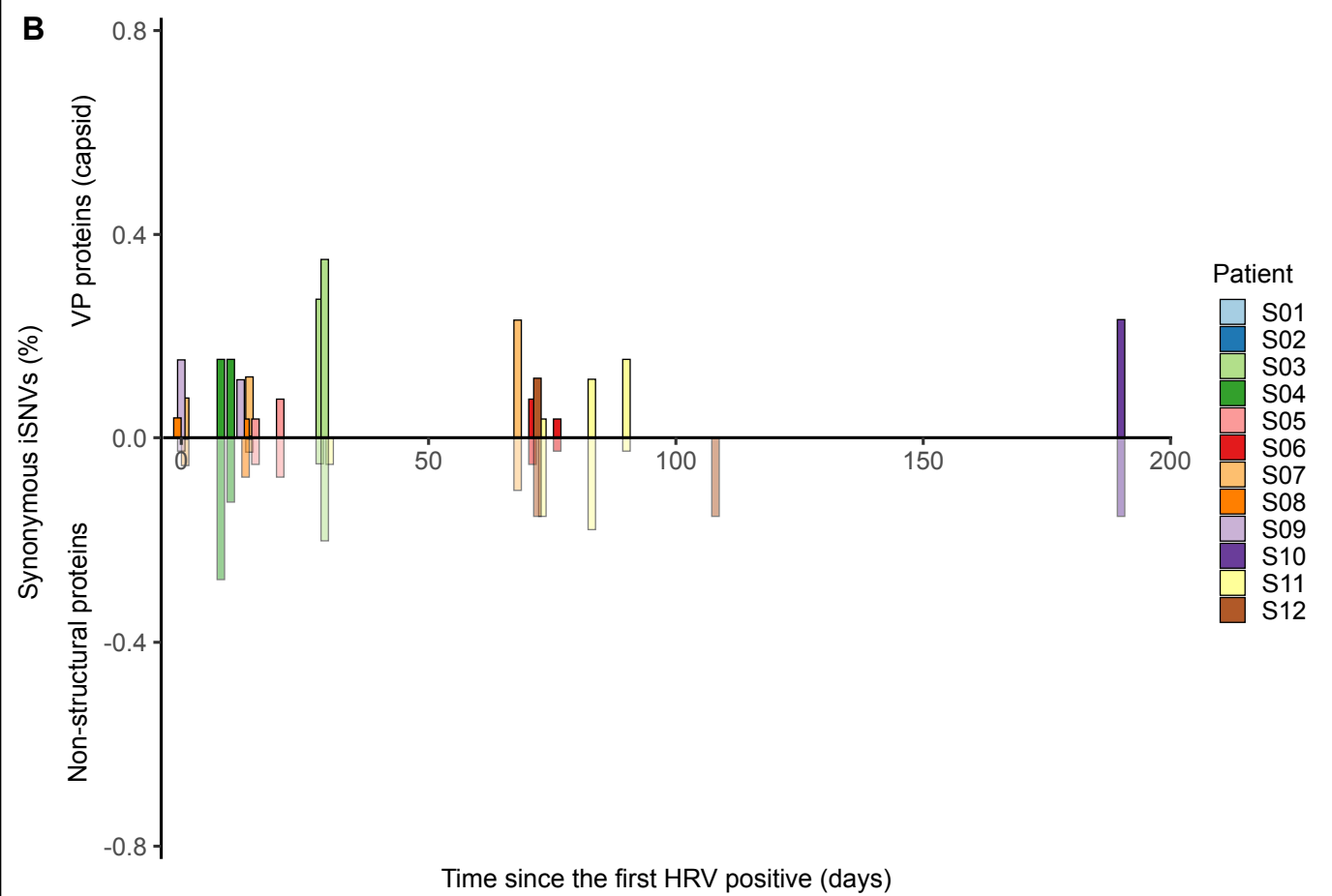

Supplement: Supplement 10 — Supplemental Figure 6. Non-synonymous and synonymous iSNVs percentage in the capsid and the non-structural proteins. The percentage of sites in the capsid proteins (positive values) and the non-structural proteins (negative values) where an iSNV was detected per sample per day of collection is shown, for both iSNVs causing a non-synonymous (A) and synonymous (B) mutation. Samples are colored by individual including a 50% transparency effect for the non-structural protein results. [file media-10.pdf]

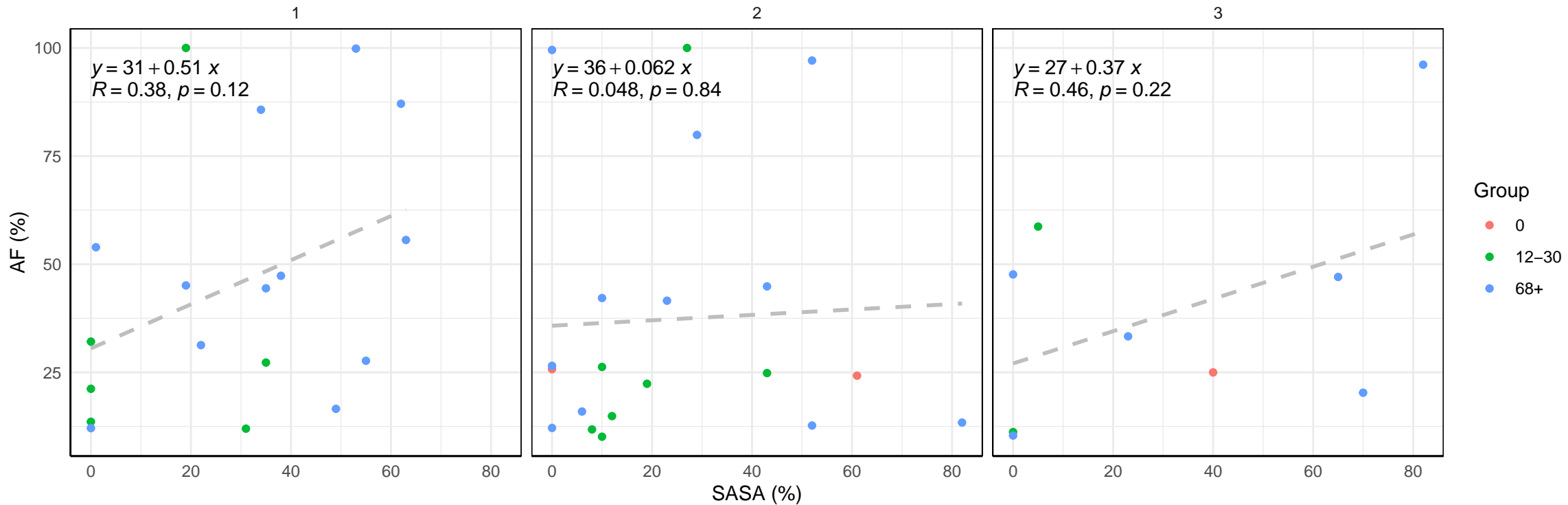

Supplement: Supplement 11 — Supplemental Figure 7. Allele frequency for each iSNV compared to solvent accessibility surface area for the corresponding VP1, VP2 and VP3 residue in HRV-A16 capsid (PDB: 1AYM). Comparison of minor allele frequency (AF) in percentage and the solvent accessibility surface area (SASA) in percentage for each iSNV located in each of the capsid proteins of HRV-A samples. Colors in dots differentiate the time categorization used in Figure 3. Regression lines are shown for each protein, together with the estimated equation and the square ratio value. [file media-11.pdf]

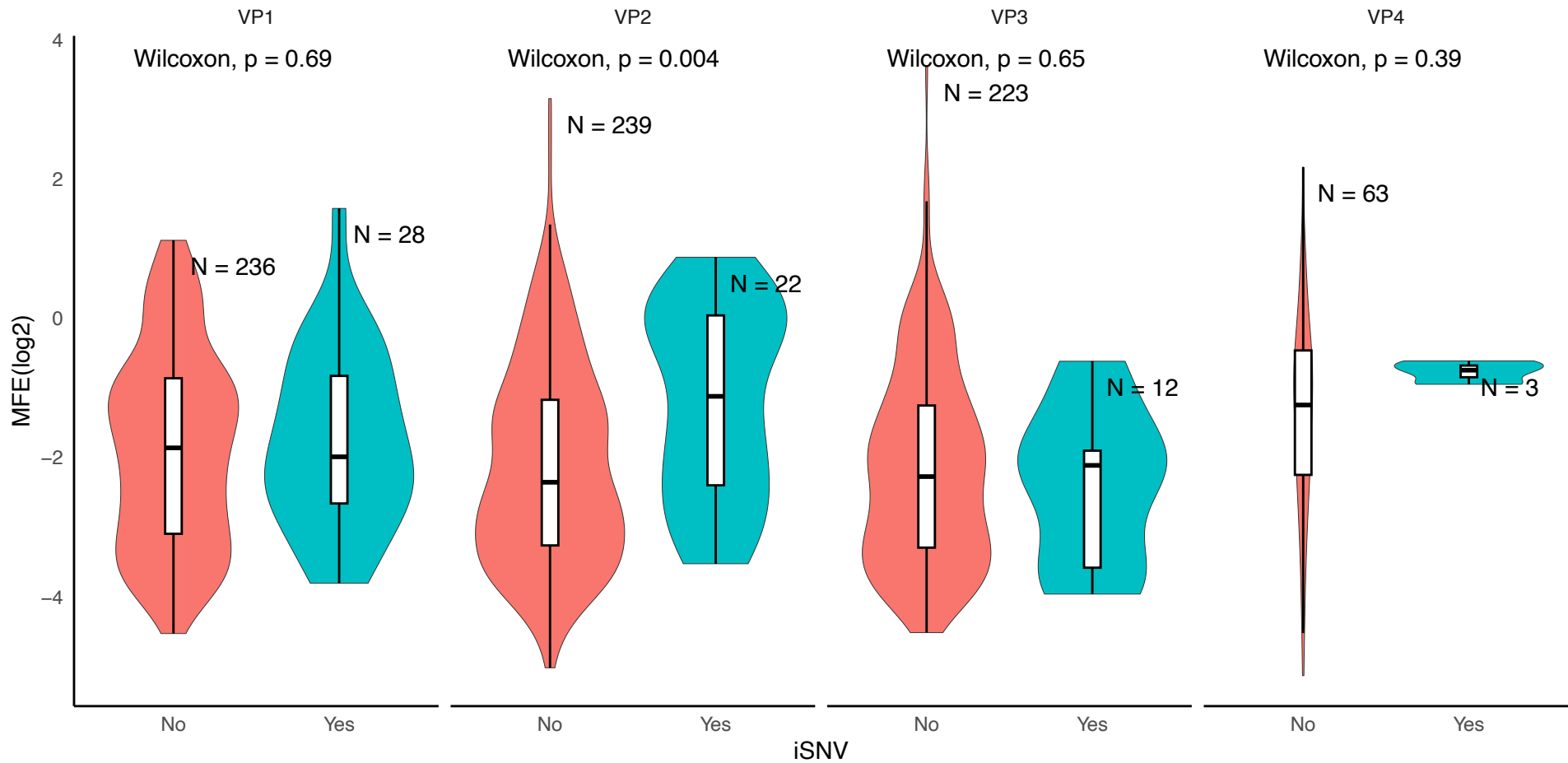

Supplement: Supplement 12 — Supplemental Figure 8. Evaluation of the mutational fitness effect in a CVB3 capsid (PDB: 4GB3) HRV-A, B, and C iSNVs. Violin plot of the average mutational fitness effect (MFE) in sites with detection of non-synonymous iSNVs in the VP1, VP2, VP3 and VP4 capsid proteins across all 12 HRV infections. P-value of the ranked Wilcoxon test is displayed. [file media-12.pdf]

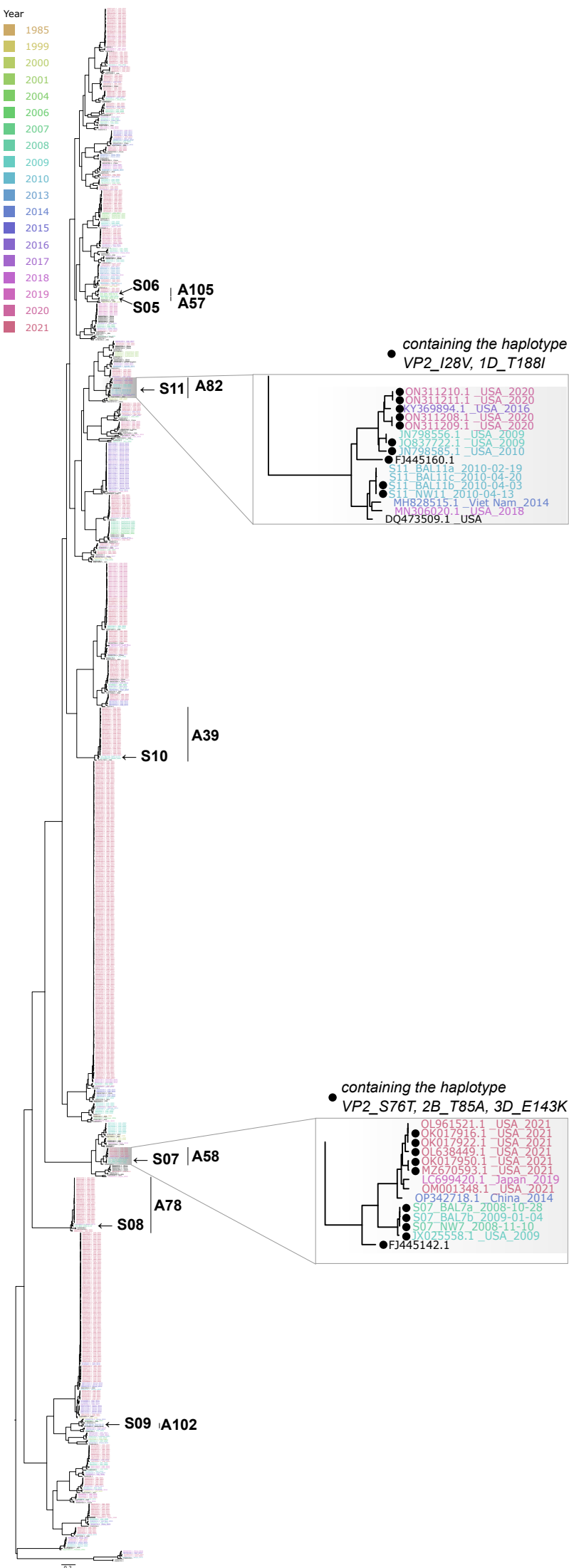

Supplement: Supplement 13 — Supplemental Figure 9. Maximum likelihood inference of HRV-A complete genome alignment including all sequences published in NCBI GenBank up to January 2023. The HRV serotypes in which classified the studied sequences are informed. Color in tip names denotes the year of collection of the sample. Sequences from the analyzed individuals are indicated with an arrow. Phylogenetic clades of serotypes containing sequences sharing the high-frequency haplotype of each individual’s samples are zoomed, and those sequences are denoted with a black circle. [file media-13.pdf]
